# The utility of B-type natriuretic peptide in the diagnosis of heart failure in the emergency department: a systematic review

**DOI:** 10.1186/1471-227X-7-6

**Published:** 2007-06-26

**Authors:** Deborah Korenstein, Juan P Wisnivesky, Peter Wyer, Rhodes Adler, Diego Ponieman, Thomas McGinn

**Affiliations:** 1 Division of General Internal Medicine, Department of Medicine, Mount Sinai School of Medicine, New York, USA; 2 Pulmonary Division, Department of Medicine, Mount Sinai School of Medicine, New York, USA; 3 Emergency Medicine Residency Program, New York Presbyterian Hospital, New York, USA

## Abstract

**Background:**

Dyspnea is a common chief complaint in the emergency department (ED); differentiating heart failure (HF) from other causes can be challenging. Brain Natriuretic Peptide (BNP) is a new diagnostic test for HF for use in dyspneic patients in the ED. The purpose of this study is to systematically review the accuracy of BNP in the emergency diagnosis of HF.

**Methods:**

We searched MEDLINE (1975–2005) supplemented by reference tracking. We included studies that reported the sensitivity and specificity of BNP for diagnosing HF in ED patients with acute dyspnea. Two reviewers independently assessed study quality. We pooled sensitivities and specificities within five ranges of BNP cutoffs.

**Results:**

Ten studies including 3,344 participants met inclusion criteria. Quality was variable; possible verification or selection bias was common. No studies eliminated patients with obvious medical causes of dyspnea. Most studies used the Triage BNP assay; all utilized a clinical reference standard. Pooled sensitivity and specificity at a BNP cutoff of 100–105 pg/ml were 90% and 74% with negative likelihood ratio (LR) of 0.14; pooled sensitivity was 81% with specificity of 90% at cutoffs between 300 and 400 pg/ml with positive LR of 7.6.

**Conclusion:**

Our analysis suggests that BNP has moderate accuracy in detecting HF in the ED. Our results suggest utilizing a BNP of less than 100 pg/ml to rule out HF and a BNP of greater than 400 pg/ml to diagnose HF. Many studies were of marginal quality, and all included patients with varying degrees of diagnostic uncertainty. Further studies focusing on patients with diagnostic uncertainty will clarify the real-world utility of BNP in the emergency management of dyspnea.

## Background

Heart failure (HF) is a major public health problem in the United States whose incidence has been rising in recent years [1,2]. Twelve to 15 million office visits and 6.5 million hospital days are attributable to HF each year, and HF is responsible for more Medicare expenditure than any other diagnosis[3].

While common, HF can be difficult and time consuming to diagnose, with no available single definitive test. Brain Natriuretic Peptide (BNP) is released by the ventricle in response to increased volume or pressure, and has been noted to be elevated in patients with HF, offering promise as a quantitative diagnostic test for the disease. Beginning in the mid-1990s, BNP has been evaluated as a diagnostic test for HF in various settings, including primary care and urgent care. In more recent years its use has become widespread in emergency departments in the evaluation of acute dyspnea. While the FDA and other organizations in North America and Europe have recommended its inclusion in the workup of HF, others have commented that the utility of BNP is not yet clear from the available literature[4,5].

We conducted a systematic review of the literature to summarize the available evidence about the sensitivity and specificity of brain natriuretic peptide in the diagnosis of heart failure in the emergency department.

## Methods

We used systematic methods to identify relevant studies, determine study eligibility, evaluate study methodological quality, and summarize findings regarding diagnostic accuracy [6-9].

### Data sources and study eligibility

We conducted a literature search of MEDLINE (1975–2005) using combinations of the key words diagnosis, heart failure, congestive heart failure, BNP, natriuretic peptide or peptides, and dyspnea. The search was constructed to retrieve articles which included the key words "CHF", "heart failure" or "congestive heart failure", along with "BNP", "natriuretic peptide" or "natriuretic peptides", and either "dyspnea" or "diagnosis" and was limited to human subjects. We augmented our computerized literature search by manually reviewing the reference lists of identified studies and of other published reviews [5,10,11]. We included articles published in any language.

Two investigators independently evaluated potential articles to decide if they were eligible for inclusion in the systematic review. Disagreements were resolved by discussion. Studies were considered eligible for inclusion if they 1) addressed the usefulness of BNP to quantify the probability of HF among patients presenting with acute dyspnea to an emergency department setting 2) included at least 50 patients and if 3) the absolute numbers of true-positive, false-negative, true-negative, and false-positive observations were available or derivable for the data presented. Articles assessing the accuracy of N-terminal probrain natriuretic peptide (NT-proBNP) were excluded, as NT-proBNP represents a distinct diagnostic test with its own performance characteristics. We attempted to contact authors of potential articles to provide any missing information. In cases of articles with overlapping study subjects, we chose either the article with more complete information or the article with the larger number of included participants.

HF is a clinical syndrome which can be challenging to diagnose, and there is no single test which serves as the criterion standard for its diagnosis [10,12]. We therefore included studies with a clinical criterion standard, in which the diagnosis of HF was determined by one or more expert cardiologists with access to all clinical information, including assessment of ventricular function and symptomatic response to treatment.

### Assessment of study quality

The methodological quality of the selected studies was assessed, and data were abstracted independently by 2 reviewers (DK and TM). We assessed study quality using the QUADAS[13]method. We utilized an adapted checklist which excluded items for which articles had been pre-selected or which were not applicable. Discrepancies were resolved by consensus.

Variables extracted included the study design, the number of patients with and without HF, characteristics of the study population (age distribution, gender, and ethnicity), clinical variables evaluated and whether they were clearly defined, reference standard utilized, and the diagnostic performance of BNP.

The optimal design for assessing the accuracy of a diagnostic test is considered to be a prospective blind comparison of the test and the reference standard in a consecutive series of patients from a relevant clinical population[6,14]. To assess the quality of the studies included in the meta-analysis data were collected regarding the study design, reference standard, adequacy of patient spectrum, description of the reasons for patient withdrawals, and the presence of verification or selection bias. An adequate description of the spectrum of patients included in a study can help clinicians know whether to generalize the results to their patients. We considered that a study met this criterion if the following information was provided about the study population: 1) demographics including age, gender and racial distribution, 2) proportion of patients with history of HF and either asthma or COPD, and 3) the number of patients hospitalized.

Verification bias occurs if the decision to perform the reference test is based on the result of the test under study. For instance, if patients with positive, as opposed to negative, BNP preferentially receive the criterion standard evaluation, the sensitivity of the test can be falsely elevated because of the incorrect exclusion of false negatives from the analysis. In cases in which not all patients were subjected to the reference test, the study was scored as having verification bias[15].

Selection bias can be present when not all patients presenting with the relevant condition are included in order of entry (consecutive) into the study, or when this selection is not random. If it was not clear from the text that a consecutive series of patients was included or a random subset, the corresponding study was scored as non-consecutive.

### Statistical analysis

To evaluate agreement between raters for the assessments of study quality, we calculated the observed percentage agreement and the κ coefficient for interrater reliability[16].

The results of diagnostic tests with numerical results can be conceived not as dichotomously "positive" or "negative," but in a continuous fashion, in which different results may impact pre-test probability to different degrees. Under these circumstances interval likelihood ratios (LR) can be calculated for ranges of values; for example, there might be a LR associated with a BNP between 100 and 200 pg/ml which reflects the impact on pre-test probability of a BNP greater than 100 but less than 200. The raw data required to calculate interval LRs was not available to us for all studies. Data was only available to calculate pooled LRs. Data at different dichotomous BNP cutoffs; i.e. for a BNP greater than or less than a particular value. We grouped these cutoffs into ranges and pooled data from any study that reported sufficient data to construct a 2 by 2 table for a cutoff value within that range.

For each study, we recorded the true-positive rate (sensitivity) and the true negative rate (specificity) for various cutoff values of BNP as provided by each study. The heterogeneity of all indexes was evaluated using a homogeneity test based on the χ^2 ^test. A result of p < 0.1 was considered significant given the relatively low power of this test. In addition, the *I*^2 ^statistic was calculated to assess the impact of heterogeneity on the results. This statistic describes the percentage of the variability in effect estimates that it is due to heterogeneity rather than sampling error (chance). A *I*^2 ^value of 0% represents no heterogeneity and 25, 50, and 75% indicate low, moderate, and high heterogeneity[17]. We planned to pool data regardless of heterogeneity so that clinicians could have an overall estimate of the performance of this diagnostic test, although heterogeneity may limit the validity of the pooled estimate.

We pooled data from different studies by grouping their results within five ranges of BNP cutoffs in pg/ml: 50–80, 100–105, 150–199, 200–299 and 300–400. For each range of BNP cutoff, we included data from any study that reported sensitivity and specificity for BNP values in that range. When studies reported results for more than one cutoff in the range, we used the cutoff which was closest to the middle of the range. We utilized the Random Effects Model to calculate pooled LRs for each BNP cutoff[18]. These LRs can be used to interpret values greater than or less than a BNP in each cutoff range; they can not be utilized to precisely interpret a BNP value within each cutoff range.

To assess publication bias, we created a funnel plot of individual study log odds ratios plotted against a function of the sample size as described by Deeks et al [19]. We tested for funnel plot asymmetry based on a regression of standardized effect estimates against precision to evaluate whether the intercept deviates from zero[20].

Statistical analyses were performed using SAS version 9.0 (SAS Institute, Cary, NC).

## Results

### Selection of studies

**Figure 1:**
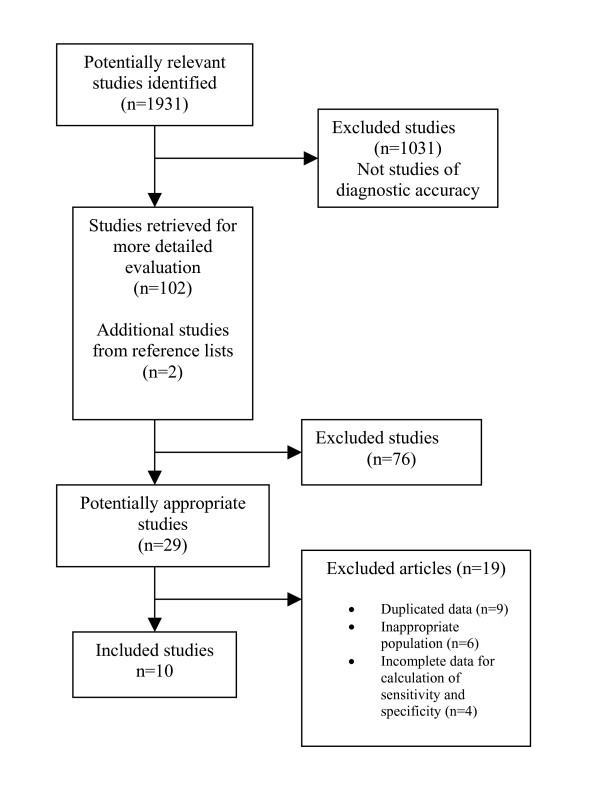
Flow of studies in the review.

Figure 1 outlines the flow of article retrieval for the review. Of the ten included articles, nine were published in English and one was published in French[21]. Data from the French article was extracted by one of the investigators (DK) who speaks French, and was excluded from the kappa calculation. Several studies reported results for overlapping patient populations [21-32]. Eight studies reported results from the BNP Multinational Study[23,24,28-31,33,34]. To avoid duplication, we retained the report in the New England Journal of Medicine [29] as it included the most complete description of demographics and BNP performance characteristics. The patients reported on in the study by Dao[25] et al were a subset of the patients in the study by Morrison et al[5,32], so we retained only the Morrison report. The two publications by Ray[35,36] reported on the same set of patients, so we retained the larger report. Of the ten studies in the analysis, none eliminated patients with obvious HF or lung disease. Many studies included incomplete reports of demographic and other information. The characteristics of the studies analyzed are outlined in Table 1[21,26,29,32,36-42].

**Table 1:** Characteristics of the studies included in the review

**Study Year**	**# subjects**	**Age (mean +/- SD)**	**% male**	**History of HF (%)**	**History of asthma or COPD (%)**	**Patients admitted (%)**	**BNP assay used**	**Final diag-nosis HF (%)**	**Cutoff (pg/ml)**	**Sensi-tivity (%)**	**Speci-ficity (%)**
Jourdain 2002	125	72†	*	*	*	100	Triage	72	300	94	86
Logeart 2002	163	67 ± 15	67	49	*	90	Triage	71	100	96	31
Maisel 2002	1,586	64 ± 17	56	33	41	72	Triage	47	100	90	76
Morrison 2002	321	*	95	42	40	43	Triage	42	105	86	94
Villacorta 2002	70	72 ± 16	47	37	44	*	Triage	51	200	100	97
Lainchbury 2003	205	70 ± 14	49	25	42	*	Triage	34	100	77	84
Knudsen 2004	155	76†	45	*	47	*	Triage	48	100	90	55
Ray 2004	308	80 ± 8	50	20	25	100	Triage	46	100	90	59
Mueller 2005	251	73†	93	65	*	*	AxSYM BNP	55	100	96	61
Alibay 2005	160	80 ± 14	48	38	34	58	Triage	38	150	94	61

Sensitivity and Specificity are presented for the BNP value closest to 100 pg/ml from each study.

* Not reported † Standard deviation not reported

The ten studies in the systematic review included 3,344 participants in North America, South America, New Zealand and Europe. The mean age of participants ranged from 64 to 80 years and between 45% and 93% of the participants were men. Nine of the ten studies utilized the Triage BNP Assay[26,29,32,36-40], which is widely used and commercially available. The other used the AxSYM BNP assay[41](table 1). In seven studies the expert opinion of two or more cardiologists with access all available clinical data served as the reference standard for the diagnosis of HF; three studies[21,40,41] utilized the opinion of only one cardiologist.

The spectrum of patients appears to be quite broad in most studies. Most included primarily older patients; gender distribution varied widely. Between 43 and 100% of enrolled patients were admitted to the hospital. Since hospital admission criteria are often subjective and may vary across health care systems, these rates may be due to practice variation or to variability in disease severity across the studies. Patients with obvious trauma as a cause of dyspnea were excluded from all studies, while all studies included patients with an obvious medical cause (i.e. asthma exacerbation, COPD or HF) of their symptoms. Thus, the patients included in our analysis represent a population of patients presenting to the emergency department with dyspnea, with varying degrees of diagnostic uncertainty.

### Methodological quality and study characteristics

The observed inter-rater agreement for assessment of study quality was κ = .88[30]. The results of the quality assessment of the included studies are listed in table 2. Only one of the studies (10%) clearly defined its spectrum of patients[29]. In all ten studies the diagnosis of HF was determined without knowledge of the BNP (blinding). Selection bias was clearly absent in six studies (60%). The presence of verification bias was difficult to ascertain in most of the studies, but was possible in three (30%). One study (10%) had unexplained patient withdrawals[21]. No study reported inter-reader reliability for the reference standard (kappa), and no evaluated study met all quality criteria.

**Table 2:** Quality measures of included studies

**Study, Year**	**Were important inclusions and exclusions explained?**	Was patient spectrum clearly defined?*	**Was the diagnosis of HF determined without knowledge of the BNP result?**	**Was agreement reported for the reference standard?**	**Was selection bias present?**	**Was verification bias present?**	**Were withdrawals explained?**
Jourdain 2002	Yes	No	Yes	No	No	No	No
Logeart 2002	Yes	No	Yes	No	No	No	Yes
Maisel 2002	Yes	Yes	Yes	No	Possible	No	N/A†
Morrison 2002	Yes	No	Yes	No	Yes	No	N/A†
Villacorta 2002	Yes	No	Yes	No	No	No	N/A†
Lainchbury 2003	No	No	Yes	No	Possible	Possible	N/A†
Knudsen 2004	Yes	No	Yes	No	No	Possible	N/A†
Ray 2004	Yes	No	Yes	No	Possible	No	Yes
Alibay 2005	Yes	No	Yes	No	No	No	N/A†
Mueller 2005	Yes	No	Yes	No	No	No	Yes

*Information available on age, gender, race, prevalence of a HF history and history of asthma and/or COPD, and the percentage of patients admitted to the hospital.

A funnel plot did not suggest evidence of publication bias.

### Diagnostic accuracy

The sensitivity and specificity of BNP for diagnosing HF varied (Table 1), and statistically significant heterogeneity was present (p < .05, *I*^2 ^> 0.5). Many studies reported sensitivity and specificity of BNP using the cutoff value with the greatest combined sensitivity and specificity, while many reported sensitivity and specificity using a cutoff at or near 100 pg/ml, which has become the conventional threshold for a positive test. Sensitivity at or near a BNP cutoff of 100 pg/ml ranged from 86% to 96%. Specificity at this cutoff ranged from 31% to 94%. Pooled negative LR for this range was .14 by the random effects model (Table 3). Sensitivity was not appreciably higher at lower cutoffs in most studies[26,29,38,39]. Several studies evaluated performance characteristics at higher BNP cutoffs, up to 400. At BNP cutoffs of 150–199 pg/ml[26,29,32,36,37,39,41], sensitivity was between 85–94% and specificity ranged widely from 45–85%. BNP cutoff values between 200 and 299 pg/ml had sensitivities between 78 and 100% and specificities between 66 and 97%[26,32,36,39,41]. Only 3 studies[21,36,39] evaluated cutoffs above 299; cutoffs of 300–400 pg/ml had sensitivity of 67–94% and specificity between 84 and 92%, with a pooled positive LR of 7.6 (Table 3). Pooled LRs for each range of cutoff values are shown in table 3.

**Table 3:** Pooled LRs for BNP in emergency department settings

**BNP cutoff (pg/ml)**	**Number of studies/number of patients with information on this cutoff**	**Positive LR**	**Negative LR**
50–80	4/2109	2.4	.08
100–105	7/2989	3.4^‡^	.14^‡^
150–199	8/2944	3.8	.16
200–299	6/1268	4.6	.16
300–400	3/596	7.6^‡^	.17^‡^

^‡^LR from random effects model

## Discussion

The current study presents a systematic review of the literature on the accuracy of B-type natriuretic peptide for diagnosing HF in the emergency department. Our review differs from others which have addressed this issue. The review by Schwam[5] described the individual studies in detail but did not systematically assess their quality. A recent review by Wang et al[10] performed quality assessments but did not report all aspects of study quality. A review by Doust et al[11] performed quality assessments and pooled data across studies, but calculated a pooled diagnostic odds ratio, which is not directly applicable to clinical practice. As the use of BNP has become increasingly common, we feel there is a need for quality assessed pooled data for clinical application. Our review is the first to present a complete quality assessment of the included articles and to pool data in a way that is relevant to practicing clinicians.

A BNP of 100 pg/ml is often cited as the best cutoff for the diagnosis of HF[43]. We found a pooled positive LR of 3.4 and negative LR of .14. At higher cutoffs of between 300 and 400 pg/ml, the positive LR rises to 7.6, with a negative LR of .17. Thus, our analysis suggests that among adult patients who with suspected heart failure, a low BNP seems to make HF unlikely, and very high BNP makes HF likely. BNP values between 100 and 300 pg/ml may not be helpful in diagnosing HF.

Table 4 demonstrates the utility of BNP in diagnosing HF in the context of varying pre-test probabilities. With a pre-test probability of 10% or 30%, a BNP of < 105 pg/ml results in a probability of HF of 2% or 5% respectively and is clinically useful in ruling out HF. A higher pre-test probability of 70% coupled with the same low BNP results in a 25% chance of HF, which is less definitive and may result in further diagnostic testing. A very high pre-test probability of 90% and a low BNP results in a 56% likelihood of HF. Similarly, a diagnosis of HF can be made in a patient with a high (70% or 90%) pre-test probability and an elevated BNP of > 300 pg/ml; while an elevated BNP in a patient with a low (30%) pre-test probability leads to a 77% chance of HF and might result in further diagnostic testing.

**Table 4:** Impact of high and low BNP results on pre-test probabilities

**Pre-test probability**	**Post-test probability for BNP < 105 pg/ml**	**Post-test probability for BNP > 300 pg/ml**
10%	2%	46%
30%	5%	77%
50%	12%	88%
70%	25%	95%
90%	56%	99%

Several limitations of the evaluated studies should be considered. First, there were potential methodological issues including possible selection bias in many of the studies and verification bias in a few. No studies reported inter-relater agreement for the reference standard, and a few studies failed to clarify important inclusion and exclusion criteria. Most important, however, may be the issue of spectrum. In studies of diagnostic tests, it is important that investigators enroll patients in whom there is diagnostic uncertainty [44], in whom the test would be used in clinical practice. The studies of BNP recruited broadly, and likely included patients in whom there was diagnostic uncertainty in addition to patients in whom the diagnosis was clear. For example, some patients presenting with dyspnea certainly had obvious asthma exacerbations, and these patients were included in the studies of BNP although they are not patients in whom a BNP assay would be utilized in clinical practice. The broadness of the spectrum in this case may have resulted in a biased estimation of the accuracy of the diagnostic test[44,45]. This potential bias limits the applicability of the included studies and of our analysis to clinical practice. Some investigators explored the impact of this broad spectrum by performing sub-group analysis looking only at patients in whom there was diagnostic uncertainty. In the Breathing Not Properly study, BNP did perform well in the subset of patients with a history of pulmonary disease[30] with sensitivity and specificity of 93% and 77% at a cutoff of 100 pg/ml, similar to that of the larger population. This finding suggests that accuracy of BNP as determined by the studies overall may in fact reflect the accuracy of the test in patients in whom there is a high degree of diagnostic uncertainty. We look forward to a confirmation of these findings in other populations.

The best evidence in support of the widespread use of a new diagnostic test is a randomized trial demonstrating that its use improves quality of care. [46,47] In the case of BNP, such a trial has already been published. In the B-type Natriuretic Peptide for Acute Shortness of Breath Evaluation (BASEL) Study[48] Mueller and colleagues performed a single-blind randomized trial in Switzerland in which patients presenting the emergency department with acute dyspnea were assigned to a diagnostic strategy including a single bedside BNP measurement or a "standard" diagnostic strategy which excluded BNP. As in the studies included in our analysis, patients with obvious trauma were excluded. A total of 452 patients were randomized; 58% were men and the mean age was 71 years. Clinicians were given guidelines for the interpretation of BNP values. A BNP level of < 100 pg/ml indicated that HF was unlikely and other causes of dyspnea should be investigated while a BNP > 500 pg/ml made HF the most likely diagnosis. No strong conclusions about HF were recommended for patients with values between 100 and 500 pg/ml. The study found lower rates of hospital admission (75% vs. 85%), significant reductions in time to discharge (8 vs. 11 days) and an $1850 reduction in cost in the group randomized to BNP testing. In-hospital and 30-day mortality were not significantly different in the two groups.

On initial inspection these strongly positive results seem inconsistent with our findings that BNP is only a moderately accurate diagnostic test. In fact, some have criticized the BASEL study [49]. Patients enrolled in the study received somewhat scripted care, which may have included testing that is not routinely performed [50]. The specific nature of the care of patients in each group and the diagnostic tests they received was not described in the publication [48]. The severity of illness in the study group is also striking. Although presenting symptoms seemed moderate, 80% of enrolled patients were hospitalized, of whom 20% were admitted to the intensive care unit, and the median length of stay in the control group was 11 days. While these characteristics may be related to the standards of practice in Switzerland or to the particulars of the study, it is unclear whether the population evaluated is representative of the broad group of patients presenting with dyspnea to emergency departments.

It is difficult, however, to disregard the dramatic finding of the BASEL study, and some of the utility of BNP may have been related to the way in which it was interpreted in the study. The study utilized two separate diagnostic cutoffs for BNP: a level of 100 pg/ml to rule out HF and a level of 500 pg/ml to rule in HF. Our determinations of the performance characteristics of BNP support the use of two diagnostic cutoffs. The pooled likelihood ratio (LR) was 0.14 for a BNP of < 105 pg/ml (pooled sensitivity 90% and specificity 74%). A LR of < 0.1 can generally rule out disease independent of pre-test probability and a LR of > 10 can similarly rule in disease independent of pre-test probability [44]. LRs of between 0.1 and 0.2 or between 5 and 10 have moderate ability to rule out or rule in disease [44]. The LR of 0.14 indicates that a BNP of < 105 will moderately lower the pre-test probability of HF, and may be clinically useful. The performance of a BNP cutoff of 500 pg/ml has not been studied; the highest cutoff we found in the literature was 399. In this highest cutoff range of 300–400 pg/ml, the pooled LR for a positive test, or elevated BNP, is 7.6, which represents moderate ability to rule in HF. It is possible that using a higher cutoff of 500 would perform better. Clearly, though, a lower cutoff to rule out HF and a higher cutoff to rule in HF is supported by the evidence, and has in fact been adopted in practice guidelines [51].

Our study has some methodological limitations. We pooled data from different studies with obvious heterogeneity, utilizing the random effects model to determine pooled LRs. We opted to perform pooling despite the heterogeneity because we believe that there is a clinical need for a compilation of findings regarding BNP, so that clinicians may understand its utility in clinical practice. We utilized the random effects model to minimize the bias associated with heterogeneous results, but the validity of the pooled estimates may still be limited by the presence of heterogeneity. In addition, we were limited by the quality of the available data, which was not ideal.

## Conclusion

In summary, our analysis suggests that BNP performs moderately as a diagnostic test for HF in the emergency department, with very high and very low values contributing significantly to making a diagnosis. Most of the studies recruited a wide spectrum of patients presenting with dyspnea, including patients with diagnostic uncertainty as well as patients with more apparent diagnoses in whom a diagnostic test might not be used in clinical practice. In order to clarify the true performance of this diagnostic test among patients in whom it is needed, studies which include only patients with diagnostic uncertainty should be performed. Until then, BNP can be best utilized by interpreting separate cutoffs for ruling in HF and ruling out HF. The BASEL study provides good evidence of the utility of BNP in a more severely ill population in Switzerland, but its findings may not be generalizable to different populations.

## Abbreviations

BNP Brain natriuretic peptide

COPD Chronic obstructive pulmonary disease

ED Emergency department

HF Heart failure

LR Likelihood ratio

NTproBNP N-terminal probrain natriuretic peptide

pg/ml picograms per milliliter

## Competing interests

The author(s) declare that they have no competing interests.

## Authors' contributions

DK participated in the design of the study, performed searches, reviewed articles, performed quality assessments and drafted the manuscript. JPW participated in the design of the study and performed the statistical analysis. PW participated in the design of the study and edited the manuscript. RA and DP performed searches and reviewed articles. TM participated in the design of the study, performed quality assessments and edited the manuscript. All authors have read and approved the final manuscript.

## Pre-publication history

The pre-publication history for this paper can be accessed here:


